# Posterior Tibial Slope Is Highly Variable and Asymmetric in Asian Osteoarthritic Knees: A Three-Dimensional CT Analysis

**DOI:** 10.3390/jcm15114123

**Published:** 2026-05-27

**Authors:** Sun Ho Cha, Min Jae Lee, Ho Jung Jung, Ji Hyo Hwang, Joong Il Kim

**Affiliations:** Department of Orthopaedic Surgery, Kangnam Sacred Heart Hospital, Hallym University College of Medicine, Seoul 07441, Republic of Korea; chash5521@naver.com (S.H.C.); mjlee951224@gmail.com (M.J.L.); hodge.jung@gmail.com (H.J.J.); hwangjihyo36@gmail.com (J.H.H.)

**Keywords:** posterior tibial slope, robotic-assisted total knee arthroplasty, knee alignment, tibial slope asymmetry, personalized TKA

## Abstract

**Purpose**: Posterior tibial slope (PTS) is an important anatomical parameter that influences knee kinematics and clinical outcomes following total knee arthroplasty (TKA). Recent advances in CT-based robotic-assisted TKA (RA-TKA) have enabled accurate three-dimensional (3D) evaluation of knee morphology. However, limited data are available regarding PTS and its associated coronal and sagittal alignment factors in Asian patients with knee osteoarthritis. The purpose of this study was to quantify medial and lateral PTS and to identify alignment factors associated with PTS, thereby clarifying the relevance of individualized 3D assessment for TKA planning. **Methods**: This retrospective study evaluated 236 knees from 236 Asian patients with knee OA undergoing primary RA-TKA (MAKO^®^, Stryker, Kalamazoo, MI, USA). Radiological parameters, including medial posterior tibial slope (MPTS), lateral posterior tibial slope (LPTS), medial proximal tibial angle (MPTA), lateral distal femoral angle, mechanical hip–knee–ankle angle, arithmetic hip–knee–ankle angle, and femur flexion angle were automatically calculated by MAKO planning software based on predefined anatomical landmarks and reference axes. Correlation and multivariate regression analyses were performed to assess the relationships between PTS and other coronal, sagittal alignment parameters. **Results**: The cohort showed an overall varus tendency, with a mean mechanical HKA angle of −8.3° ± 5.21°, while coronal alignment itself was not used as an exclusion criterion. PTS demonstrated substantial variability among individuals as well as between the medial and lateral sides within the same knee. The distribution of PTS values showed a wide range, with MPTS ranging from −4.8° to 24.5° and LPTS ranging from −1.4° to 17.4°. The MPTS was significantly greater than the LPTS (7.85° ± 4.72° vs. 6.33° ± 4.04°, *p* < 0.001). No statistically significant association was observed between MPTS and MPTA. Multivariate linear regression demonstrated that LPTS was the strongest positive predictor of MPTS (β = 0.365, *p* < 0.001), while height (β = −0.169, *p* = 0.006) and femoral flexion angle (β = −0.195, *p* < 0.001) were significant negative predictors. **Conclusions**: In Asian patients with knee OA, PTS showed substantial inter-individual variability and significant medial–lateral asymmetry on 3D CT analysis. These findings suggest that a fixed PTS target may not fully reflect patient-specific native morphology, and further outcome-based studies are needed to define the optimal compartment-specific PTS reference for TKA planning.

## 1. Introduction

Posterior tibial slope (PTS) is a fundamental anatomical parameter in total knee arthroplasty (TKA), playing a pivotal role in post-operative knee kinematics, flexion gap balance, and posterior cruciate ligament (PCL) tension [1,2,3]. Inadequate restoration of PTS has been associated with reduced range of motion, poorer clinical outcomes and early aseptic loosening [1,3]. Specifically, a recent study demonstrated that restoring the native PTS within a 4° threshold is crucial; deviations beyond this threshold can lead to inferior clinical outcomes after cruciate-retaining (CR) TKA [4]. Therefore, accurate preoperative assessment of the native PTS and its appropriate restoration are critical prerequisites for achieving optimal functional outcomes and patient satisfaction following TKA.

Despite its profound clinical importance, accurately measuring the native PTS remains a challenge. Historically, most previous studies and routine clinical practices have relied on two-dimensional (2D) lateral radiographs to evaluate PTS. However, 2D measurements are highly susceptible to projection errors, rotational variations in the lower limb during image acquisition, and the superimposition of the medial and lateral tibial plateaus [5,6,7]. More importantly, 2D radiography inherently fails to reflect the complex three-dimensional (3D) morphologic variations in the tibial plateau, particularly the well-recognized asymmetry between the medial and lateral compartments [8].

Recent advances in imaging technology and the widespread adoption of robotic-assisted TKA (RA-TKA) have enabled highly accurate, 3D computed tomography (CT)-based evaluations of knee morphology. These 3D techniques eliminate the rotational errors of 2D radiographs and allow for independent, compartment-specific analysis of the tibial plateau [7]. However, despite the availability of these advanced tools, limited data exists regarding the comprehensive 3D evaluation of compartment-specific PTS (medial versus lateral) and their associated anatomical factors in advanced osteoarthritic knees.

This lack of detailed morphological data is particularly problematic in Asian populations. Asian patients with advanced knee osteoarthritis (OA) commonly exhibit distinct morphological characteristics, including a higher prevalence of severe varus alignment and pronounced proximal tibial vara, compared with Western populations [9,10,11]. These unique ethnic and morphological phenotypes can significantly influence the geometry of the tibial plateau and alter the characteristics of the PTS. Given that most contemporary TKA implants and surgical guidelines were primarily designed based on the morphometric data of Caucasian populations, applying a “one-size-fits-all” approach to PTS, regardless of individual native morphology in Asian patients, may lead to suboptimal clinical outcomes.

Therefore, the primary purpose of this study was to quantitatively evaluate the medial and lateral posterior tibial slopes (MPTS and LPTS) using 3D CT-based bone models in Asian patients with advanced knee OA. The secondary purpose was to identify specific coronal and sagittal alignment factors that are associated with PTS. We hypothesized that significant asymmetry exists between the MPTS and LPTS and that the magnitude of the PTS is correlated with specific lower limb alignment parameters.

## 2. Materials and Methods

### 2.1. Patient Selection

**Figure 1:**
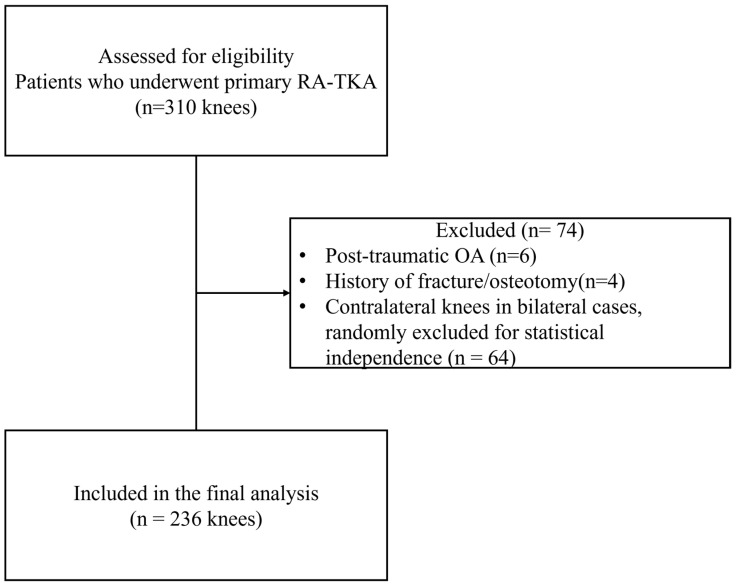
Flow diagram of the patient selection process in accordance with the Strengthening the Reporting of Observational Studies in Epidemiology (STROBE) guidelines.

An institutional database with prospectively collected data from patients who underwent primary RA-TKA (MAKO, Stryker) for knee OA between July 2023 and January 2026 was retrospectively reviewed. In accordance with the STROBE guidelines, a participant selection flow diagram was constructed (Figure 1). Patients with post-traumatic OA or a history of fracture or previous osteotomy on the ipsilateral knee were excluded. Coronal alignment was not used as an exclusion criterion. Therefore, knees with varus, neutral, and valgus coronal alignment were eligible for inclusion. The coronal alignment profile of the final cohort was described using the preoperative mechanical hip–knee–ankle angle (mHKA) and Coronal Plane Alignment of the Knee (CPAK) classification. To maintain statistical independence, when both knees from the same patient were eligible, one knee was randomly selected using a computer-generated sequence and the contralateral knee was excluded. Finally, 236 knees from 236 patients were included in the final analysis. This study was conducted in accordance with the principles of the Declaration of Helsinki and was approved by the Institutional Review Board of our hospital; the requirement for informed consent was waived due to the retrospective design and use of de-identified data.

### 2.2. CT Acquisition and 3D Modeling

All patients underwent preoperative CT scans according to the manufacturer-recommended protocol for the robotic-assisted system. The scans included the hip, knee, and ankle joints to assess the mechanical axis of the lower limb, with a slice thickness of 0.6 mm. The CT data were segmented and reconstructed into 3D bone models using the proprietary software of the robotic system. Anatomical landmarks, mechanical axes, and joint reference planes were automatically identified and calculated by the system’s proprietary planning software. Therefore, the measurements represented osseous morphology derived strictly from the segmented CT-based bone models, and no manual measurement of PTS was performed by observers. The 3D models and landmark positions were routinely reviewed during the preoperative planning process, and cases with inadequate CT quality or segmentation failure were excluded. Furthermore, because osteophytes and irregular degenerative contours may inherently influence the reconstructed osseous surface, the measured values in this study should be interpreted as CT-based “bony slope” rather than cartilage- or meniscus-inclusive “functional slope”.

### 2.3. Radiological Measurements

**Figure 2:**
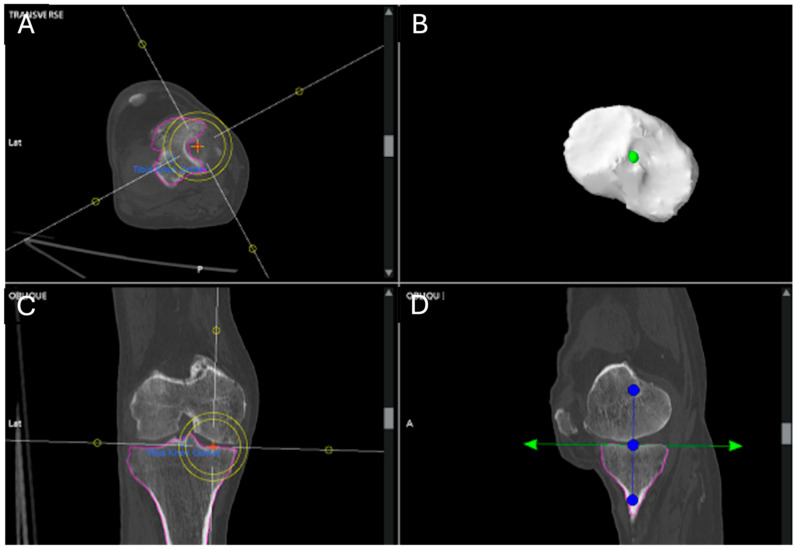
3D CT-based measurement of the posterior tibial slope (PTS) using the robotic planning software. The screen displays four corresponding views used to define the anatomical landmarks and axes: (**A**) transverse view, (**B**) 3D bone model, (**C**) coronal view, and (**D**) sagittal view. In the sagittal view (**D**), the medial posterior tibial slope (MPTS) is determined automatically. The blue dots define the tibial mechanical axis, and the green line represents the perpendicular reference axis used to calculate the slope.

Radiological parameters, including MPTS, LPTS, medial proximal tibial angle (MPTA), lateral distal femoral angle (LDFA), mHKA, arithmetic HKA (aHKA), and femoral flexion angle (anatomical axis (AA)–mechanical axis (MA) sagittal), were automatically calculated by the MAKO planning (Mako Total Knee 2.0 software) based on predefined anatomical landmarks and reference axes (Figure 2). The MPTS and LPTS were measured in the sagittal plane. The slope was defined as the angle between a line tangent to the lowest point of the medial or lateral articular surface and a plane perpendicular to the tibial mechanical axis. Positive values indicated a posterior slope, whereas negative values indicated an anterior slope. The mechanical HKA angle was defined as the coronal angle between the femoral and tibial mechanical axes, with negative values indicating varus alignment and positive values indicating valgus alignment. The femoral flexion angle was defined as the angle between the AA and MA of the femur in the sagittal plane; positive values indicated extension and negative values indicated flexion. This approach is consistent with previous CT-based studies demonstrating that standardized 3D calculations, including automated measurements utilized by the MAKO planning software, can reduce observer-dependent variability in landmark selection [5,6,7,12].

### 2.4. Statistical Analysis

Statistical analysis was performed using SPSS software (version 30.0.0.0; IBM Corp., Armonk, NY, USA). The primary outcome was MPTS. Secondary outcomes included LPTS, the medial–lateral PTS difference, and the associations between MPTS and coronal or sagittal alignment parameters. The normality of continuous variables was assessed using the Shapiro–Wilk test. An a priori power analysis using G*Power software (version 3.1.9.7) [13] determined that a minimum of 138 knees was required for multiple linear regression (f^2^ = 0.15, α = 0.05, power = 0.95). Thus, our sample of 236 knees provided sufficient statistical power. Descriptive statistics were presented as means ± standard deviations (SD) for continuous variables and as frequencies (percentages) for categorical variables. For the paired comparison between MPTS and LPTS, the distribution of paired differences was assessed before applying the paired t-test. Because certain variables violated the assumption of normality, Spearman’s rank correlation coefficient (ρ) was used to analyze relationships between alignment parameters. The strength of correlation was interpreted according to Cohen’s criteria [14]: 0.1–0.3, weak; 0.3–0.5, moderate; and >0.5, strong. To identify independent predictors of MPTS, multivariate linear regression was performed using variables that showed potential association in univariate analysis or were considered clinically relevant, including height, LPTS, MPTA, LDFA, and AA–MA sagittal. Residual plots were inspected to assess linearity, homoscedasticity, and influential outliers. A *p*-value < 0.05 was considered statistically significant.

## 3. Results

**Figure 3:**
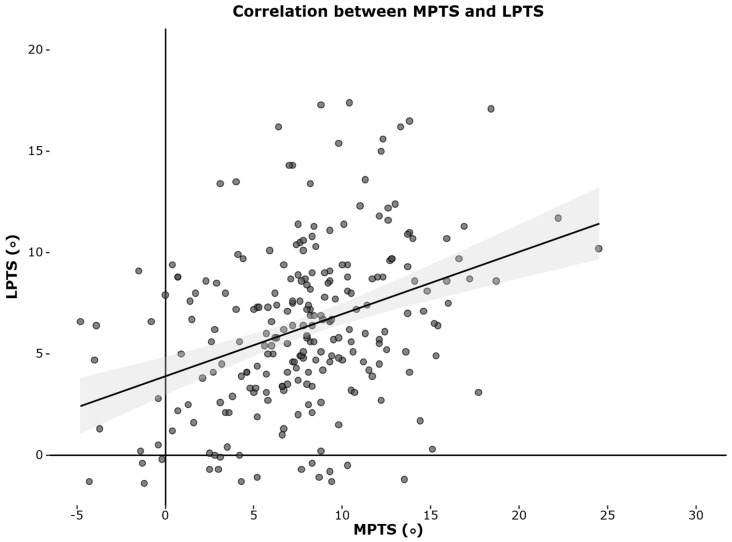
Moderate positive correlation between the medial posterior tibial slope (MPTS) and the lateral posterior tibial slope (LPTS) using Spearman’s rank correlation analysis (*p* < 0.001, ρ = 0.352).

The demographic and baseline radiographic characteristics of the 236 patients are summarized in Table 1 and Table 2. Coronal alignment was not used as an exclusion criterion; therefore, varus, neutral, and valgus knees were included in the final cohort. Overall, the cohort showed a varus tendency, with a mean preoperative mechanical HKA angle of −8.3° ± 5.21°. The mean MPTS was 7.85° ± 4.72° (range, −4.8° to 24.5°), and the mean LPTS was 6.33° ± 4.04° (range, −1.4° to 17.4°). MPTS was significantly greater than LPTS (mean difference, 1.51° ± 4.98°; *p* < 0.001) (Table 3). Spearman’s rank correlation analysis demonstrated a positive correlation between MPTS and LPTS (ρ = 0.352, *p* < 0.001) (Figure 3). No significant correlation was observed between MPTA and MPTS (ρ = 0.005, *p* = 0.938). Multivariate linear regression analysis was performed to identify factors associated with MPTS. The model included height, LDFA, MPTA, femoral flexion angle, and LPTS. Femoral flexion angle (β = −0.195, *p* < 0.001) and height (β = −0.169, *p* = 0.006) showed significant negative associations with MPTS. LPTS was the strongest positive predictor of MPTS (β = 0.365, *p* < 0.001). The regression model explained 19.4% of the variance in MPTS (adjusted R^2^ = 0.194), with no evidence of multicollinearity (variance inflation factor range, 1.020–1.082) (Table 4).

## 4. Discussion

The most important finding of this study is that PTS demonstrated marked inter-individual variability and consistent medial–lateral asymmetry in Asian osteoarthritic knees when evaluated using 3D CT analysis. MPTS was significantly greater than LPTS across the cohort, but the degree of asymmetry varied widely among individual patients, indicating that PTS is a patient-specific morphological parameter rather than a uniform anatomical constant. The lack of correlation between MPTA and MPTS suggests that coronal tibial alignment should not be assumed to reflect sagittal tibial alignment. LPTS emerged as the strongest independent predictor of MPTS; however, the relatively low explanatory power of the model indicates that MPTS cannot be reliably inferred from LPTS alone.

A key finding of this study was the significant difference between the MPTS and LPTS. MPTS was consistently greater than LPTS, with a mean difference of approximately 1.5°. This discrepancy raises a clinical question regarding which PTS (medial, lateral, or a composite value) should be referenced during surgical planning for TKA. Historically, the LPTS has often been used as a reference to avoid excessive bony resection of the lateral plateau that may occur when the steeper MPTS is used as a cutting guide [15]. However, recent 3D CT-based studies have challenged this traditional approach by highlighting substantial intra-individual variability in PTS [16,17]. In this context, strict reliance on the LPTS may not adequately reflect patient-specific medial compartment morphology particularly in CR TKA where medial stability plays a central role [18]. These observations support a more individualized approach, including consideration of independent medial and lateral PTS during preoperative planning. Although restoration of native PTS within a 4° boundary has been associated with favorable outcomes after CR TKA [4], it remains unclear which PTS should be prioritized and whether a specific boundary can be applied across compartments, underscoring the need for further studies to define the optimal reference and acceptable range of PTS restoration in relation to clinical outcomes. Therefore, the present morphological findings should be interpreted as a rationale for future outcome-based research rather than as definitive surgical guidance.

The anatomical relationship between coronal alignment (e.g., MPTA) and sagittal morphology (e.g., PTS) remains a subject of ongoing debate in the orthopaedic literature. A recent large-scale 3D CT study by Hiyama et al. [7] involving an Asian population reported that PTS is independent of coronal plane alignment. In contrast, Meier et al. [17] used 3D CT data from osteoarthritic knees and found a significant positive correlation between MPTA and MPTS. Furthermore, a cadaveric anatomical study by Foos et al. [19] reported an association between coronal plane deformity and PTS. In the present study, no significant correlation was observed between MPTA and MPTS. This finding indicates that PTS cannot be inferred from coronal alignment; therefore, PTS should be assessed independently using dedicated sagittal or 3D imaging during preoperative planning [5,6,7,16,17].

Although several studies have reported that PTS varies according to demographic factors such as ethnicity and sex [20,21], detailed morphometric data focusing specifically on Asian populations remain limited. Moreover, most previous morphometric studies have evaluated PTS as a single global parameter without distinguishing between the medial and lateral compartments [7,22], despite the recognized anatomical asymmetry of the tibial plateau. Consequently, the predictors of compartment-specific PTS in Asian populations with knee OA have not been clearly established. In the present study, LPTS emerged as the strongest predictor of MPTS. Height and femoral flexion angle also showed significant negative associations with MPTS, suggesting that patient-specific body size and sagittal femoral morphology may contribute to medial slope variability. However, because direct biomechanical evidence explaining these associations remains limited, these findings should be interpreted cautiously and regarded as hypothesis-generating. The regression model accounted for only 19.4% of the variance. Other unmeasured anatomical or biomechanical factors, such as rotational alignment, tibial torsion, and joint line obliquity, may contribute to compartment-specific PTS variability.

This study has several limitations. First, although the study specifically focused on Asian patients with advanced osteoarthritic knees undergoing CT-based RA-TKA, this design may limit generalizability to other ethnic groups, non-varus phenotypes, or non-robotic TKA workflows. Second, although all measurements were automatically generated from CT-based robotic planning software, independent validation of the automated landmark detection and segmentation process was not performed in the present study. Osteophytes, subchondral remodeling, and irregular degenerative contours may influence the reconstructed osseous surface. Third, anterior cruciate ligament (ACL) status was not routinely evaluated. Because ACL integrity has been associated with tibial plateau wear patterns in varus osteoarthritic knees, its potential influence on posterior plateau remodeling and MPTS could not be assessed [23]. Fourth, CT-based measurements reflect osseous slope and do not account for cartilage thickness or meniscal geometry; therefore, the functional slope experienced during weight-bearing may differ from the bony slope measured in this study. In advanced OA, asymmetric cartilage loss and meniscal degeneration may alter the functional joint surface, particularly in the medial compartment. Therefore, medial–lateral differences in osseous PTS may not fully correspond to functional joint kinematics [24,25]. Finally, this was a morphological imaging study and did not include postoperative kinematics, patient-reported outcomes, or implant survivorship. Future prospective studies are needed to determine whether restoring native compartment-specific slopes within defined boundaries improves knee kinematics and clinical outcomes after TKA.

## 5. Conclusions

In Asian patients with knee OA, PTS demonstrated substantial inter-individual variability and significant medial–lateral asymmetry on 3D CT analysis. MPTS was not associated with coronal tibial alignment and could not be reliably inferred from LPTS alone. These findings underscore the need for individualized preoperative assessment of MPTS and LPTS; however, further outcome-based studies are needed to determine which PTS reference is most clinically relevant and the extent of PTS restoration required to achieve optimal outcomes after TKA.

## Figures and Tables

**Table 1:** Demographic data.

Variable	N = 236 ^a^
Age, years	71.17 ± 6.93
Sex, male:female	41:195
Body mass index, kg/m^2^	26.34 ± 3.64
Right:Left	119:117
Kellgren-Lawrence grade, 3:4	72:164

^a^ Values are presented as mean ± SD or No.

**Table 2:** Preoperative radiological data.

Variable	N = 236 ^a^
MPTA, deg	84.39 ± 2.86
LDFA, deg	88.12 ± 2.80
mHKA, deg ^b^	−8.3 ± 5.21
aHKA, deg ^c^	−3.73 ± 4.32
AA-MA coronal, deg ^d^	5.87 ± 0.70
AA-MA sagittal, deg ^e^	0.35 ± 3.56
MPTS, deg	7.85 ± 4.72
LPTS, deg	6.33 ± 4.04
PCA-sTEA, deg ^f^	3.03 ± 2.05
CPAK classification	
Type I	146 (61.9%)
Type II	54 (22.9%)
Type III	12 (5.1%)
Type IV	17 (7.2%)
Type V	4 (1.7%)
Type VI	1 (0.4%)
Type IX	2 (0.8%)

CPAK, coronal plane alignment of the knee; MPTA, medial proximal tibial angle; LDFA, lateral distal femoral angle; aHKA, arithmetic hip-knee-ankle angle; AA, anatomical axis; MA, mechanical axis; MPTS, medial posterior tibial slope; LPTS, lateral posterior tibial slope; PCA, posterior condylar axis; sTEA, surgical transepicondylar axis. ^a^ Values are presented as mean ± SD or No; ^b^ a positive value: valgus; a negative value: varus; ^c^ a positive value: valgus; a negative value: varus; ^d^ a positive value: valgus; a negative value: varus; ^e^ a positive value: extension; a negative value: flexion; ^f^ a positive value: external rotation; a negative value: internal rotation.

**Table 3:** Comparison of posterior tibial slope between the medial and lateral compartments.

	Mean ± SD (°)	Range (°)	Mean Differences ± SD (°)	*p* Value *
MPTS	7.85 ± 4.72	−4.8 to 24.5	1.51 ± 4.98	<0.001
LPTS	6.33 ± 4.04	−1.4 to 17.4		

MPTS, medial posterior tibial slope; LPTS, lateral posterior tibial slope; SD, standard deviation. * *p* value was calculated using a paired *t*-test. Statistically significant values are defined as *p* < 0.05.

**Table 4:** Multiple linear regression analysis of factors correlated with the MPTS.

Independent Variables	Multivariate Regression ^a^					
	Non-Standardized Coefficient		Standardized Coefficient	95% CI	VIF	*p* Value
	B	SE	ꞵ			
*Height*	*−0.121*	*0.043*	*−0.169*		*1.044*	*0.006*
*LDFA*	*0.017*	*0.104*	*0.010*		*1.082*	*0.872*
MPTA	−0.054	0.100	−0.033		1.049	0.589
*Femur flexion angle*	*−0.259*	*0.079*	*−0.195*		*1.020*	*<0.001*
*LPTS*	*0.427*	*0.070*	*0.365*	[*0.289*, *0.566*]	*1.034*	*<0.001*

B, Non-standardized Coefficient; SE, Standard Error; ꞵ, Standardized Coefficient; CI, Confidence Interval; VIF, Variance Inflation Factor; LDFA, lateral distal femur angle; MPTA, medial proximal tibia angle; LPTS, lateral posterior tibial slope. ^a^ Adjusted R^2^: 0.194, Durbin-Watson: 2.070, *p*-value = 0.001. Italicized values indicate statistical significance.

## Data Availability

The data that support the findings of this study are available on request from the corresponding author. The data are not publicly available due to privacy or ethical restrictions.

## Abbreviations

PTS	posterior tibial slope
TKA	total knee arthroplasty
RA-TKA	robotic-assisted total knee arthroplasty
MPTS	medial posterior tibial slope
LPTS	lateral posterior tibial slope
MPTA	medial proximal tibial angle
LDFA	lateral distal femoral angle
aHKA	arithmetic hip–knee–ankle angle
PCA	posterior condylar axis
PCL	posterior cruciate ligament
OA	osteoarthritis
CT	computed tomography

## References

[1] Lee H.Y., Kim S.J., Kang K.T., Kim S.H., Park K.K. (2012). The effect of tibial posterior slope on contact force and ligament stresses in posterior-stabilized total knee arthroplasty: Explicit finite element analysis. Knee Surg. Relat. Res..

[2] Okazaki K., Tashiro Y., Mizu-uchi H., Hamai S., Doi T., Iwamoto Y. (2014). Influence of the posterior tibial slope on the flexion gap in total knee arthroplasty. Knee.

[3] Seo S.S., Kim C.W., Moon S.W. (2013). Clinical results associated with changes of posterior tibial slope in total knee arthroplasty. Knee Surg. Relat. Res..

[4] Cho Y.T., Jung H.J., Kim J.I. (2025). Restoring native posterior tibial slope within 4° leads to better clinical outcomes after cruciate-retaining robot-assisted total knee arthroplasty with functional alignment. Knee Surg. Sports Traumatol. Arthrosc..

[5] Hecker A., Lerch T.D., Egli R.J., Liechti E.F., Klenke F.M. (2021). The EOS 3D imaging system reliably measures posterior tibial slope. J. Orthop. Surg. Res..

[6] Seki K., Seki T., Siigi E., Imagama T., Yamabe T., Sakai T. (2023). Comparing inter- and intraobserver reliability between two-dimensional radiographic and three-dimensional CT measurements. Acta Orthop. Belg..

[7] Hiyama S., Rao R.P., Xie F., Takahashi T., Takeshita K., Pandit H. (2025). Comparative analysis of posterior tibial slope measurements: Accuracy and reliability of radiographs and CT. J. Orthop..

[8] Hashemi J., Chandrashekar N., Gill B., Beynnon B.D., Slauterbeck J.R., Schutt R.C., Mansouri H., Dabezies E. (2008). The geometry of the tibial plateau and its influence on the biomechanics of the tibiofemoral joint. J. Bone Jt. Surg. Am..

[9] Harvey W.F., Niu J., Zhang Y., McCree P.I., Felson D.T., Nevitt M., Xu L., Aliabadi P., Hunter D.J. (2008). Knee alignment differences between Chinese and Caucasian subjects without osteoarthritis. Osteoarthr. Cartil..

[10] Ho J.P.Y., Merican A.M., Ayob K.A., Sulaiman S.H., Hashim M.S. (2021). Tibia vara in Asians: Myth or fact? Verification with three-dimensional computed tomography. J. Orthop. Surg..

[11] Moon Y.W., Park J.H., Lee S.S., Kang J.W., Lee D.H. (2022). Distal femoral phenotypes in Asian varus osteoarthritic knees. Knee Surg. Sports Traumatol. Arthrosc..

[12] Wong W.K., Zulkhairi S.Z., Chua H.S. (2025). CT-Based Software-Generated Measurements Permit More Objective Assessments of Arithmetic Hip-Knee-Ankle Axis and Joint Line Obliquity. Life.

[13] Faul F., Erdfelder E., Lang A.G., Buchner A. (2007). G*Power 3: A flexible statistical power analysis program for the social, behavioral, and biomedical sciences. Behav. Res. Methods.

[14] Cohen J. (1988). Statistical Power Analysis for the Behavioral Sciences.

[15] Kuwano T., Urabe K., Miura H., Nagamine R., Matsuda S., Satomura M., Sasaki T., Sakai S., Honda H., Iwamoto Y. (2005). Importance of the lateral anatomic tibial slope as a guide to the tibial cut in total knee arthroplasty in Japanese patients. J. Orthop. Sci..

[16] Calek A., Hochreiter B., Hess S., Amsler F., Leclerq V., Hirschmann M.T., Behrend H. (2022). High inter- and intraindividual differences in medial and lateral posterior tibial slope are not reproduced accurately by conventional TKA alignment techniques. Knee Surg. Sports Traumatol. Arthrosc..

[17] Meier M., Janssen D., Koeck F.X., Thienpont E., Beckmann J., Best R. (2021). Variations in medial and lateral slope and medial proximal tibial angle. Knee Surg. Sports Traumatol. Arthrosc..

[18] Hirschmann M.T., Muller W. (2015). Complex function of the knee joint: The current understanding of the knee. Knee Surg. Sports Traumatol. Arthrosc..

[19] Foos J., Amakoutou K., Cooperman D.R., Liu R.W. (2023). A cadaveric anatomical study of the relationship between proximal tibial slope and coronal plane deformity. J. Knee Surg..

[20] Clinger B.N., Plaster S., Passarelli T., Marshall J., Wascher D.C. (2022). Differentiation in Posterior Tibial Slope by Sex, Age, and Race: A Cadaveric Study Utilizing 3-Dimensional Computerized Tomography. Am. J. Sports Med..

[21] Koh Y.-G., Nam J.-H., Chung H.-S., Chun H.-J., Kim H.-J., Kang K.-T. (2020). Morphometric study of gender difference in osteoarthritis posterior tibial slope using three-dimensional magnetic resonance imaging. Surg. Radiol. Anat..

[22] Meric G., Gracitelli G., Aram L., Swank M.L., Bugbee W.D. (2015). Tibial slope is highly variable in patients undergoing primary total knee arthroplasty: Analysis of 13,546 computed tomography scans. J. Arthroplast..

[23] Moschella D., Blasi A., Leardini A., Ensini A., Catani F. (2006). Wear patterns on tibial plateau from varus osteoarthritic knees. Clin. Biomech..

[24] Hohmann E., Tetsworth K., Glatt V., Ngcelwane M., Keough N. (2021). The posterior horn of the medial and lateral meniscus both reduce the effective posterior tibial slope: A radiographic MRI study. Surg. Radiol. Anat..

[25] Wen D., Bohlen H., Mahanty S., Wang D. (2025). Posterior Tibial Slope Measurements of the Medial and Lateral Plateaus Vary Widely Between Magnetic Resonance Imaging and Computed Tomography. Arthroscopy.

